# Insights into transcription factors controlling strawberry fruit development and ripening

**DOI:** 10.3389/fpls.2022.1022369

**Published:** 2022-10-10

**Authors:** Carlos Sánchez-Gómez, David Posé, Carmen Martín-Pizarro

**Affiliations:** Departamento de Mejora Genética y Biotecnología, Instituto de Hortofruticultura Subtropical y Mediterránea (IHSM), Universidad de Málaga - Consejo Superior de Investigaciones Científicas, Departamento de Biología Molecular y Bioquímica, Facultad de Ciencias, UMA, Málaga, Spain

**Keywords:** strawberry, *Fragaria vesca*, *Fragaria* × *ananassa*, transcription factors, development, ripening

## Abstract

Fruit ripening is a highly regulated and complex process involving a series of physiological and biochemical changes aiming to maximize fruit organoleptic traits to attract herbivores, maximizing therefore seed dispersal. Furthermore, this process is of key importance for fruit quality and therefore consumer acceptance. In fleshy fruits, ripening involves an alteration in color, in the content of sugars, organic acids and secondary metabolites, such as volatile compounds, which influence flavor and aroma, and the remodeling of cell walls, resulting in the softening of the fruit. The mechanisms underlying these processes rely on the action of phytohormones, transcription factors and epigenetic modifications. Strawberry fruit is considered a model of non-climacteric species, as its ripening is mainly controlled by abscisic acid. Besides the role of phytohormones in the regulation of strawberry fruit ripening, a number of transcription factors have been identified as important regulators of these processes to date. In this review, we present a comprehensive overview of the current knowledge on the role of transcription factors in the regulation of strawberry fruit ripening, as well as in compiling candidate regulators that might play an important role but that have not been functionally studied to date.

## Introduction

Fleshy fruit ripening is an extremely complex process that involves biochemical, physiological and structural changes resulting in fruits more appealing for seed dispersal. Among these changes, fruit ripening involves an alteration in color, in the content of sugars, organic acids and secondary metabolites such as volatile compounds, which influence flavor and aroma, and the remodeling of cell walls, resulting in the softening of the fruit. The regulatory mechanisms underlying fruit ripening rely on the coordinated roles of phytohormones, transcription factors (TFs) and epigenetic modifications, which are in turn regulated by external and internal stimuli (Li et al., 2022b). Those regulatory mechanisms have been mainly studied for climacteric fruit ripening, in which tomato (*Solanum lycopersicum*) is the model organism. Climacteric ripening is characterized by the requirement of the phytohormone ethylene and a burst in cellular respiration, and many regulators involved in its regulation have been described so far (Klee and Giovannoni, 2011). In contrast, non-climacteric fruit ripening, for which the woodland and cultivated strawberry species (*Fragaria vesca* and *Fragaria* × *ananassa* respectively) have become the model, is not dependent on ethylene or a respiration burst but is mainly regulated by abscisic acid (ABA) (Jia et al., 2011; Bai et al., 2021).

Strawberry is a popular fruit crop thanks to its flavor, aroma and nutritional value, with a huge impact on the agricultural economy of many countries. Furthermore, its extracts are known to produce cytotoxic effects on several human cancer lines (Lucioli et al., 2019) and against ageing progression (Giampieri et al., 2017), therefore benefitting human health. Besides their importance both economically and as a health-promoting fruit, the development of strawberry fruits is also very interesting botanically. Thus, strawberries are achenetum-type fruits whose fleshy part develops from the flower receptacle, while the achenes, the real fruits, are derived from the fertilized carpels and dot the surface of the receptacle (Liu et al., 2020).

**Figure 1:**
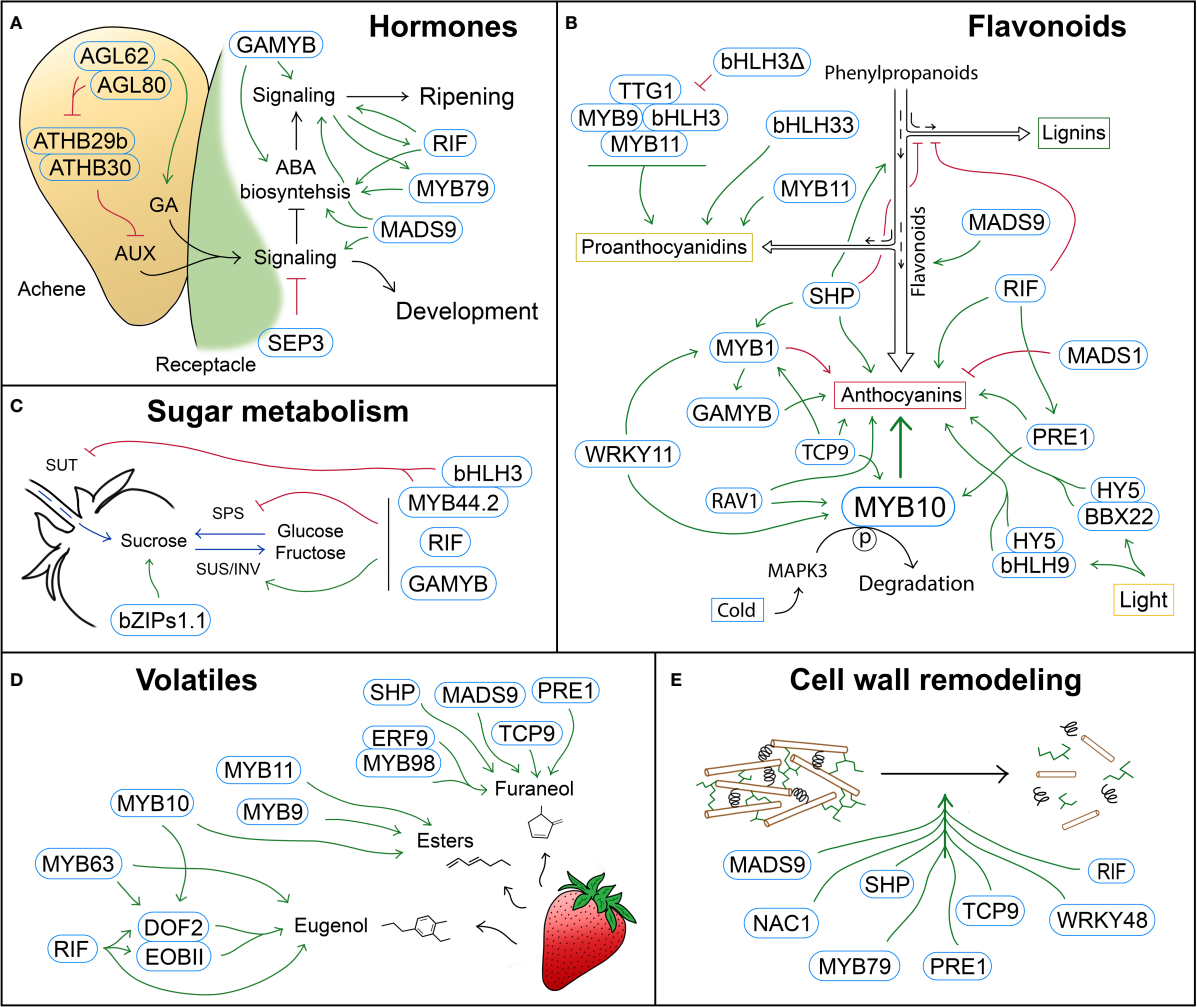
Schematic representation of TFs with a role in different strawberry ripening-related processes. **(A)** Hormone biosynthesis and signaling are regulated by complex GNRs that govern the switch from the development stages, in which auxin (AUX) and gibberellic acid (GA) promote cell fruit division and expansion, to the ripening stages, which are mainly regulated by abscisic acid (ABA). **(B)** Regulation of the phenylpropanoid pathway by TFs that control metabolic fluxes and modulate different branches of the pathway, such as the lignin, proanthocyanidin or anthocyanin pathways. **(C)** TFs involved in the regulation of sugar metabolism (SPS, SUS and INV) and transport (SUT). **(D)** TFs controlling the biosynthesis of volatile compounds, mainly esters, as well as furaneol and eugenol. **(E)** TFs regulating cell wall modification, and therefore fruit softening during ripening. Blue rounded rectangles indicate TFs. Green arrows and red block symbols denote positive and negative regulation respectively.

Besides the function of different phytohormones in the regulation of strawberry fruit ripening (Symons et al., 2012; Gu et al., 2019), the role of many TFs in strawberry ripening has been studied to date, although a comprehensive compilation of this knowledge is lacking. In this review, we will focus on how strawberry TFs regulate fruit development- and ripening-related processes like hormonal balance, flavonoids biosynthesis, carbohydrates metabolism, volatile production and cell wall modifications, which are summarized in **Table 1** and **Figure 1**.

**Table 1:** List of strawberry fruit development- and ripening-related transcription factors and their biological role.

TF Name	TF Family	Gene ID FvH4_v4.0.a2	Biological role	Regulated genes	Regulation by ABA	Regulation by ABA (Medina-Puche et al., 2016)	Regulation by auxin	References
FaRAV1	AP2/ERF	FvH4_5g19881	Phenylpropanoid biosynthesis	*CHS*, *CHI*, *F3H*, *DFR*, *ANS*, GT1, *MYB10*	+	–	nd	Zhang et al., 2020
FaERF9	AP12/ERF	FvH4_2g26630	Aroma (furaneol)	*QR*	nd	–	nd	Zhang et al., 2018b
FaBBX22	B-box	FvH4_3g17750	Phenylpropanoid biosynthesis	*PAL*, *ANS*, *F3’H*, *UFGT*, *RAP*	nd	+	nd	Liu et al., 2022
FaSPT	bHLH	FvH4_1g16230	Fruit size/shape	nd	nd	/	–	Tisza et al., 2010
FaPRE1 (HLH)	bHLH	FvH4_3g04290	Fruit development and ripening	*MYB10*, *EOBII*, *4CL*, *LAR*, *F3H*, *CHS*, *DFR*, *GST*, *UFGT*, *QR*, *EGS2*, *CAD1*, *AAT*, *PL*, *EXP*, *PG1*, *RGlyaseI*	+	–	–	Medina-Puche et al., 2021
FvbHLH9	bHLH	FvH4_1g16130	Phenylpropanoid biosynthesis	*CHS*, *DFR*, *ANS*, *UFGT*	nd	+	nd	Li et al., 2020
FabHLH3 (Schaart)	bHLH	FvH4_2g23700	Phenylpropanoid biosynthesis	*ANR*	nd	/	nd	Schaart et al., 2013; Xu et al., 2021
FabHLH3Δ (Schaart)	bHLH	FvH4_2g23700	Phenylpropanoid biosynthesis	nd	nd	/	nd	Schaart et al., 2013
FabHLH33	bHLH	FvH4_7g14230	Phenylpropanoid biosynthesis	*DFR*, *CHS2*, *UFGT*	nd	nd	nd	Schaart et al., 2013; Wei et al., 2018; Lin-Wang et al., 2014; Xu et al., 2021
FabHLH3 (Wei)	bHLH	FvH4_2g22150	Phenylpropanoid biosynthesis	*SPS3*	nd	/	nd	Wei et al., 2018
FvMYC1	bHLH	FvH4_5g02520	Phenylpropanoid biosynthesis	*DFR2*	nd	–	nd	Xu et al., 2021
HY5	bZIP	FvH4_2g29440	Phenylpropanoid biosynthesis	*CHS*, *DFR*, *ANS*, *UFGT*	nd	–	nd	Li et al., 2020
FvbZIP11	bZIP	FvH4_2g09540	Sugar metabolism	nd	nd	+	nd	Zhang et al., 2022b
FabZIPs1.1	bZIP	FvH4_5g39200	Sugar metabolism	nd	nd	+	nd	Chen et al., 2020
FvbZIP46	bZIP	FvH4_6g20610	Plant defense	*CHI2*, *CHI3*, *CHI4*	nd	/	nd	Lu et al., 2020a
FaDOF2	DOF	FvH4_2g14390	Aroma (eugenol)	*EOBII*, *EGS2*	+	+	–	Molina-Hidalgo et al., 2017
FvATHB29b	HD-Zip	FvH4_5g17830	Auxin metabolism	*YUC10*, *TAA1*	nd	/	nd	Guo et al., 2022
FvATHB30	HD-Zip	FvH4_6g48610	Auxin metabolism	*YUC10*, *TAA1*	nd	/	nd	Guo et al., 2022
FaMADS9	MADS-box	FvH4_6g46420	ABA and auxin metabolism	*MYB10*, *SHP*, *ARFs*, *IAAs*, *FaGH3.6*, *FaGH3.17*, *NCED1*, *NCED2*, *NCED3*, *CHS1*, *CHI1-3*, *F3H, RAP*, *4CL*, *FLS3*, *PAL1*, *PAL2*, *C4H*, *4CL*, *F3H*, *QR*, *PE1*, *PE2*, *PG1*, *PG2*, *PL*	nd	+	nd	Seymour et al., 2011; Vallarino et al., 2020
FaSHP	MADS-box	FvH4_6g37880	Fruit development and ripening	*MYB1*, *MYB10*, *MADS9*, *PAL*, *CHS*, *QR*, *PG1*, *PL*, *EG1*	+	+	–	Daminato et al., 2013
FaMADS1a	MADS-box	FvH4_6g46420	Fruit development and ripening	*PAL6, CHS*, *DFR*, *ANS*	–	+	+	Lu et al., 2018; Chen et al., 2022
FvSEP3	MADS-box	FvH4_4g23530	Flower and fruit development	*YUC10*, *LAX1*, *GH3.17*, *ARF7*, *LAX2*, *ARF2*, *PIN5*, *GH3.18*, *IAA20*, *GA3ox*, *GA2ox*, *GID1b*	nd	+	nd	Pi et al., 2021
FvAGL62	MADS-box	FvH4_2g03030	Auxin and GA metabolism	*YUC1*, *YUC5*, *YUC10*, *TAA1*, *TAR1*, *TAR2*, *GA20OX1c*, *GA20OX1d*, *GA3OX1a*, *GA3OX1b*, *ATHB29b*, *ATHB30*	nd	/	nd	Guo et al., 2022
FvAGL80	MADS-box	FvH4_6g08460	Auxin and GA metabolism	*ATHB29b*, *ATHB30*	nd	/	nd	Guo et al., 2022
FaMYB1/ FvMYB1/ FcMYB1	MYB	FvH4_5g17111	Phenylpropanoid biosynthesis	*CHI*, *F3H*, *DFR*, *LAR*, *ANR*, *ANS*, *UFGT*	+	+	nd	Aharoni et al., 2001; Ling-Wang et al., 2010; Paolocci et al., 2011; Salvatierra et al., 2013; Kadomura-Ishikawa et al., 2015b
FaMYB10/ FvMYB10	MYB	FvH4_1g22020	Phenylpropanoid biosynthesis	*MYB10*, *PAL*, *F3H*, *CHS*, *CHI*, *DFR*, *ANS*, *UFGT*, *RAP*, *CHS*, *UFGT*, *DFR*	+	+	–	Lin-Wang et al., 2010; Hawkins et al., 2016; Zhang et al., 2017; Luo et al., 2018; Gao et al., 2020; Castillejo et al., 2020; Wang et al., 2020; Zhang et al., 2020; Manivannan et al., 2021; Mao et al., 2022
FaEOBII	MYB	FvH4_6g50930	Aroma (eugenol)	*EGS2*, *CAD1*	+	+		Medina-Puche et al., 2015
FaGAMYB	MYB	FvH4_7g04470	ABA metabolism	*MYB1*, *MYB10*, *NCED1*, *NCED2*, *ABI5*, *DREB1*, *MYC1*, *TTG1*, *SPS1*, *SPS2*, *SPS3*, *SUS*	nd	+	nd	Vallarino et al., 2015
FaMYB44.2	MYB	FvH4_2g33810	Sugar metabolism	*MYB1*, *MYB10*, *GAMYB*, *SUS1*, *SPS1*, *SPS2*, *SPS3*, *SUT1*, *HXK2*, *TPS7*, *PYL1*, *JAZ1*, *ARF6B*	nd	/	nd	Wei et al., 2018
FvMYB79	MYB	FvH4_5g32460	ABA metabolism and cell wall remodeling	*MYB10*, *CHS*, *CHI*, *DFR*, *UFGT*, *PME38*, *PME*, *EXP*, *PL*, *PG*, *EGase*	+	+	nd	Cai et al., 2022
FaMYB63	MYB	FvH4_3g15320	Aroma (eugenol)	*MYB10*, *EOBII*, *4CL*, *PAL*, *EGS1*, *EGS2*, *CAD1*	–	/	–	Wang et al., 2022
FaMYB98	MYB	FvH4_6g51000	Aroma (furaneol)	*QR*	nd	/	nd	Zhang et al., 2018b
FaMYB9	MYB	FvH4_2g31100	Aroma (C6 volatiles)	*ANR*, *PDC*, *PDH1*, *PDH2*, *ACCase*, *MCD*, *KAR*, *KASI*, *KASII*, *LOX5*, *KCT*, *ADH*, *AAT*	nd	–	nd	Schaart et al., 2013; Lu et al., 2020b
FvMYB11/ FaMYB11	MYB	FvH4_6g34650	Phenylpropanoid biosynthesis and aroma (esters)	*CHS, DFR, PDC, PDH2, MCD, LOX5, KCT, KCT2, ADH*, *AAT*	nd	/	nd	Schaart et al., 2013; Lu et al., 2021
FvMYB3	MYB	FvH4_1g08390	Phenylpropanoid biosynthesis	*CHS*	nd	/	nd	Xu et al., 2021
FvMYB9	MYB	FvH4_2g31100	Phenylpropanoid biosynthesis	*CHS*	nd	–	nd	Xu et al., 2021
FvMYB21	MYB	FvH4_2g31080	Phenylpropanoid biosynthesis	*CHS*, *DFR*	nd	–	nd	Xu et al., 2021
FvMYB22	MYB	FvH4_2g31090	Phenylpropanoid biosynthesis	*CHS*, *DFR*	nd	–	nd	Xu et al., 2021
FvMYB41	MYB	FvH4_3g45450	Phenylpropanoid biosynthesis	*CHS*	nd	/	nd	Xu et al., 2021
FvMYB45	MYB	FvH4_4g19310	Phenylpropanoid biosynthesis	*CHS*	nd	/	nd	Xu et al., 2021
FvMYB77	MYB	FvH4_5g39550	Phenylpropanoid biosynthesis	*CHS*	nd	/	nd	Xu et al., 2021
FvMYB75	MYB	FvH4_5g34660	Phenylpropanoid biosynthesis	*DFR*	nd	+	nd	Xu et al., 2021
FvMYB64	MYB	FvH4_5g15200	Phenylpropanoid biosynthesis	*CHS*, *DFR*	nd	–	nd	Xu et al., 2021
FvMYB105	MYB	FvH4_7g16990	Phenylpropanoid biosynthesis	*CHS*, *DFR*	nd	–	nd	Xu et al., 2021
FcNAC1	NAC	FvH4_3g08490	Fruit development and ripening	*PL*	+/-	+	–	Carrasco-Orellana et al., 2018
FaRIF	NAC	FvH4_3g20700	Fruit development and Phenylpropanoid biosynthesis	*SHP*, *NAC042*, *EOBII*, *DOF2*, *SPT*, *PRE1*, *XYL3*, *PL3-4*, *GH9B15*, *ADPG2*, *EXP1-2-3*, *PME39*, *AGPs*, *PL2*, *PG1*, *PME38*, *RGlyase1*, *PAL1-2*, *C4H*, *4CL2*, *CHS1*, *HCT*, *CCR*, *CAD9*, *EGS2*, *NES1*, *NCED3-5*, *ZEP*, *HVA22*, *SnRK2.6*, *HY5*, *CYP79B*, *AIL6*, *IAA9*, *ASR*, *SUS1*, *SPS1*, *ERF17-74*	+	+	/	Moyano et al., 2018; Martín-Pizarro et al., 2021
FvTCP9	TCP	FvH4_5g12710	Fruit development and ripening	*MYB1*, *MYB10*, *NCED1*, *PYR1*, *SnRK2*, *ABI5*, *C4H*, *4CL*, *CHS*, *CHI*, *F3H*, *DFR*, *ANS*, *UFGT*, *PAL*, *QR*, *PG1*, *PL*, *EG1*	/	–	nd	Wei et al., 2016; Xie et al., 2020
FaWRKY1	WRKY	FvH4_4g23480	Plant defense	*GST*	+	–	/	Encinas-Villarejo et al., 2009
FaWRKY11	WRKY	FvH4_4g06830	Plant defense	*MYB1*, *MYB10*, *CHI2*, *CHI3*, *CHI4*, *PR1*, *PR4*	nd	–	nd	Wang et al., 2021
FvWRKY48	WRKY	FvH4_6g53770	Cell wall remodeling	*PLs*, *β-gal*, *PLA*	nd	+	nd	Zhang et al., 2022a
FaWRKY25	WRKY	FvH4_3g39850	Plant defense	*CHI2*, *CHI3*	nd	–	nd	Jia et al., 2021

The list of genes regulated by the TFs is listed, as well as if they are positively (+), negatively (-) or not (/) regulated by ABA and auxin. *nd* denotes *not determined.*

## Phytohormones in strawberry fruit development and ripening regulation

Once ovaries have been fertilized after pollination, auxin and gibberellic acid (GA) biosynthesis is initiated in achenes and transported to the receptacle. It has been recently and elegantly reported the role of the complex of type I MADS-box genes *FvAGL62/FvAGL80* promoting auxin biosynthesis in the endosperm of fertilized seeds (Guo et al., 2022). Guo and collaborators also showed that the FvAGL62/FvAGL80-mediated auxin biosynthesis is not direct *via* activation of auxin biosynthesis genes such as *FvYUC10* or *FvTAR1* but through the repression of *FvATHBs* TFs, which negatively regulate those biosynthesis genes at pre-fertilization stages. Furthermore, GA biosynthesis genes were downregulated in *fvagl62* mutants, supporting the role of *FvAGL62* in the biosynthesis of this hormone (**Figure 1A**; Guo et al., 2022). The synthesis of both auxin and GAs is maintained during their first developmental stages, where the fruit increases in width and length due to an active cell division and cell expansion (Zhou et al., 2021). In addition, in these initial stages, GA promotes ABA catabolism in the receptacle by activating the expression of the cytochrome P450 monooxygenase *FvCYP707A4a*, which catalyses ABA’s 8’-hydroxylation, thus preventing the onset of ripening before fruit development is completed (Liao et al., 2018). Later during development, auxin and GA levels decline, as well as *FvCYP707A4a* expression, allowing ABA accumulation in the receptacle and the transition between fruit growth and ripening stages (Gu et al., 2019). ABA is of great importance since it is a dominant positive regulator of strawberry fruit ripening, as it has an essential role in the synchronization of the central regulatory network controlling all ripening-related processes like alterations of texture, color, sweetness, flavor and aroma (Li et al., 2022a).

The role of other hormones during the regulation of strawberry ripening has also been studied, although their implications are not totally well understood. For example, ethylene is known to regulate the expression of genes involved in cell wall degradation and ripening-related metabolic pathways (Castillejo et al., 2004; Merchante et al., 2013), being likely involved at later stages of the ripening process due to its late accumulation pattern and the expression of genes related to its metabolism and signaling (Gu et al., 2019). However, its role does not seem critical since exogenous treatments with the ethylene inhibitor 1-methylcyclopropane (1-MCP) do not affect the common ripeness traits such as red color, sugar accumulation or acid loss (Reis et al., 2020). Cytokinins (CKs) dramatically increase their levels at the ripe stage due to changes in the expression of genes involved in their biosynthesis and catabolism, possibly implying a role during the last stages of ripening as well (Gu et al., 2019). Salicylic acid (SA), whose roles are typically associated with plant defense, also increases through ripening, although it is not clear its function in the regulation of this process (Kim et al., 2019). Methyl jasmonate (MeJA) accumulates during fruit development and drops through ripening, however, it has been reported an acceleration of the ripening upon its external application (Han et al., 2019). Finally, even though brassinosteroids (BRs) have been implicated in the regulation of ripening in other non-climacteric fruits such as grapes (Symons et al., 2006), their level decreases during strawberry fruit development, suggesting that they might play a role during early stages in strawberries instead (Symons et al., 2012).

### Regulation of hormone metabolism, transport and signaling by TFs

The ABA biosynthetic pathway can be regulated at different levels. One of the most important enzymes in the pathway is the 9-*cis*-epoxycarotenoid dioxygenases (NCEDs), which use 9-*cis*-violaxanthin and 9′-*cis*-neoxanthin as substrates to form xanthoxin in a limiting reaction. Other regulatory steps include one of the first reactions in the pathway catalyzed by zeaxanthin epoxidase (ZEP) and the ABA catabolism mediated by CYP707A (Liao et al., 2018). Recently, a NAC TF named *Ripening Inducing Factor* (*FaRIF*) has been reported to play a key role in the regulation of strawberry fruit ripening (Martín-Pizarro et al., 2021). In this sense, stable RNAi silencing lines produce fruits with a significant reduction in the ABA content, which is consistent with lower expression levels of *FaNCED3*, *FaNCED5* and *FaZEP*, as well as other ABA signaling genes, like the ABA-induced genes *FaHVA22*, *FaSnRK2*.6 and the *ELONGATED HYPOCOTYL5* (*FaHY5*), a basic leucine zipper (bZIP) TF. Furthermore, the delayed ripening progress of *FaRIF-*silenced fruits was complemented when treated with exogenous ABA, supporting a positive feedback loop regulatory mechanism between ABA and *FaRIF* in the control of the ripening process. Besides ABA, the downregulation of *FaRIF* resulted in alterations in the expressions of genes involved in the metabolism of other hormones, including auxin and ethylene biosynthetic and signaling genes (Martín-Pizarro et al., 2021).

A similar positive regulatory feedback loop has also been reported for ABA and a MYB transcription factor, *FvMYB79*, which is positively regulated by ABA. Thus, *FvMYB79* silencing decreases the ABA content and delays fruit ripening, a phenotype that could not be reverted even after ABA treatment (Cai et al., 2022). Another MYB transcription factor *FaGAMYB* has been shown to be an important regulator of strawberry fruit ripening, as it induces ABA biosynthesis by regulating *NCED1* and *NCED2*, as well as *ABI5* and *DREB1*, two TFs involved in ABA signaling. Interestingly, the regulation of many ripening-related genes by *FaGAMYB* is produced in both ABA-dependent and -independent manners, as ABA treatment in transiently *FaGAMYB*-silenced fruits is able to recover only part of the differentially expressed genes (Vallarino et al., 2015). Other TF with a role in ABA regulation include the TCP gene *FvTCP9*, whose transient overexpression and silencing result in the accumulation and reduction of the ABA content respectively due to the regulation of the expression of *NCED1* and the signaling genes *PYR1*, *SnRK2* and *ABI5* (Xie et al., 2020).

Even though studies usually focus on the possible regulatory role of a TF upon ABA, other genes have been reported to regulate other hormones. *FvSEP3* is a MADS-box TF involved in flower identity and organogenesis, as it is a member of the ABCE model. Its role during flower and fruit development in strawberry has been studied by the generation of stable EMS mutants and CRISPR/Cas9 lines, which presented sepaloid floral organs instead of petals and stamens, as well as shorter styles compared to those of the WT (Pi et al., 2021). These aberrant flowers could develop into parthenocarpic fruit, supporting a role of *SEP* genes in repressing fruit growth in strawberry. In this context, a transcriptome analysis showed that *FvSEP3* may have a role in the inhibition of the very first steps of fruit development since genes involved in auxin metabolism (*FvYUC10*, *FvGH3.17*, and *FvGH3.18*), transport (*FvLAX1* and *FvPIN5*) and signaling (*FvARF7* and *FvIAA20*) pathways were upregulated in *fvsep3* fruits. Furthermore, *fvsep3* mutation also resulted in a misregulation of GA pathway genes, such as the biosynthetic *FveGA3ox* and the catabolic *FvGA2ox* genes, as well as the receptor *FvGID1b* (Pi et al., 2021). Besides its role at early stages of fruit development, *FvSEP3* expression increases during ripening, suggesting a role in this process. Consistently, fruit ripening is delayed in *fvsep3* mutants, although how FvSEP3 regulates this process or its putative interaction with ABA is still unknown.

Another SEPALLATA MADS-box TF, *FaMADS9*, has also been shown to regulate hormone metabolism and signaling in two independent studies, contributing to strawberry fruit ripening. In the first study, a significant delay in the ripening process was achieved by the silencing of the gene (Seymour et al., 2011), while in the second work, no visible ripening phenotype was observed in their RNAi lines (Vallarino et al., 2020). These differences might be due to the specificity of the silencing. Thus, in the case of Seymour and collaborators, some related MADS-box genes were downregulated in the *FaMADS9*-RNAi fruits, such as *SEP3-, SHATTERPROOF2-* and *AGL6-like* genes. However, in the study of Vallarino and collaborators, only *FaMADS9* was downregulated in the *FaMADS9*-silenced lines out of the 34 MADS-box genes expressed in *F.* × *ananassa* fruit, while *FaSHP* was upregulated. Interestingly, the overexpression and silencing of *FaSHP* promote an up- and downregulation of *FaMADS9*, respectively (Daminato et al., 2013), suggesting that these two genes share a feedback regulatory loop. Transcriptome analysis of the stable *FaMADS9*-silenced fruits at two ripening stages revealed alterations in auxin metabolism and signaling. Specifically, *GH3* genes, nine members out of the seventeen *AUXIN RESPONSE FACTORS* (*ARFs*) genes expressed in strawberry fruits and seven *AUX/IAA*, were downregulated in both or any of the ripening stages studied. Interestingly, and despite the normal fruit appearance, *FaMADS9*-silenced fruits showed a reduction in the ABA content, which correlates with the downregulation of *FaNCED1, FaNCED2* and *FaNCED3* biosynthetic genes (Vallarino et al., 2020).

Besides the knowledge of how all these different TFs regulate hormone biosynthesis and signaling (**Figure 1A**), several studies have also reported how some TFs are in turn regulated by different hormones (**Table 1**) (Encinas-Villarejo et al., 2009; Tisza et al., 2010; Daminato et al., 2013; Medina-Puche et al., 2015; Medina-Puche et al., 2016; Molina-Hidalgo et al., 2017; Carrasco-Orellana et al., 2018; Lu et al., 2018; Moyano et al., 2018; Medina-Puche et al., 2019; Xie et al., 2020; Zhang et al., 2020; Martín-Pizarro et al., 2021; Cai et al., 2022; Wang et al., 2022), highlighting the complex regulatory network between hormones and transcriptional regulators to control strawberry fruit development and ripening.

## Regulation of the phenylpropanoid pathway

Many of the benefits that strawberry consumption contributes to human health rely on polyphenols, whose metabolic pathway has been well studied in strawberry (Fait et al., 2008; Muñoz et al., 2011; Urrutia et al., 2015). Polyphenols constitute a structurally and functionally diverse group of compounds produced through the shikimate-phenylpropanoid pathways (Bontpart et al., 2016). The end product of the shikimate pathway, the aromatic amino acid phenylalanine, is the main precursor for the biosynthesis of most polyphenols. The phenylpropanoid pathway uses phenylalanine as the initial substrate which is transformed into 4-coumaroyl-CoA by the sequential activity of the enzymes phenylalanine ammonia-lyase (PAL), cinnamate 4-hydroxylase (C4H) and 4-coumarate-CoA ligase (4CL). Then, the 4-coumaroyl-CoA is the branching point of the pathway, being the substrate of the flavonoid or monolignol pathway (Pott et al., 2020). Among all polyphenols, strawberry is especially rich in flavonoids. The first committed step in the flavonoid biosynthetic pathway is catalyzed by the chalcone synthase (CHS), which is followed by the chalcone isomerase (CHI) to produce the flavanone naringenin. This compound is subsequently converted into the different groups of flavonoid compounds, which differ in the degree of oxidation of the three-carbon bridge, and include anthocyanins, the main responsible for the red color of strawberry fruits, and proanthocyanidins (PAs or condensed tannins), their most abundant flavonoid (Buendía et al., 2010; Petrussa et al., 2013). In particular, anthocyanins are synthesized by a series of reactions catalyzed by flavanone 3-hydroxylase (F3H), flavonoid 3’-hydroxylase (F3’H), flavonoid 3’-5’-hydroxylase (F3’5’H), dihydroflavonol 4-reductase (DFR), anthocyanidin synthase/leucoanthocyanidin dioxygenase (ANS/LDOX) and UDP-glucose flavonoid 3-*O*-glucosyltransferase (UFGT). They are synthesized at the endoplasmic reticulum and later transported into the vacuole for storage mainly by glutathione S-transferases (GSTs) (Luo et al., 2018; Castillejo et al., 2020). Among the anthocyanins, pelargonidin-3-glucoside is the major pigment in strawberry receptacles, while cyanidin-3-glucoside is present in a minor content (Almeida et al., 2007; Zhao et al., 2021). Finally, for the biosynthesis of PAs, two main enzymes are involved, i.e., leucocyanidin reductase (LAR) and anthocyanidin reductase (ANR). Many TFs have been reported to be involved in the regulation of this metabolic pathway (Karlova et al., 2014). Next, we will provide an overview of their role in the biosynthesis of these polyphenolic compounds (**Figure 1B**).

### MBW complexes

The regulation of the phenylpropanoid pathway is largely regulated by MBW ternary complexes, which are constituted by the TFs R2R3MYB and bHLH, and a WD-repeat protein. The first TF characterized in strawberry was *FaMYB1* and its regulation upon anthocyanin biosynthesis (Aharoni et al., 2001). It was firstly characterized by stable overexpressing lines in *Nicotiana benthamiana* (Aharoni et al., 2001) and *Lotus corniculatus* (Paolocci et al., 2011), where it produced a misregulation of the flavonoid biosynthetic pathway. Later, this role was also found in *Fragaria chiloensis*, a strawberry species that develops white/pinkish receptacles and red achenes at the fully ripe stage of the fruits. Salvatierra and collaborators showed that the white skin phenotype of *F. chiloensis* receptacles was reverted to red pigmented fruits by the silencing of *FcMYB1*, which resulted in the repression of flavonoid biosynthetic genes such as *CHI*, *F3H*, *DFR*, *LAR* and *ANR*, and the upregulation of *ANS* and *UFGT*. Therefore, *FcMYB1* silencing is redirecting the precursors of the flavonoid pathway from the PAs branch (*LAR* and *ANR*) towards the biosynthesis of anthocyanins (*ANS* and *UFGT*), resulting in the accumulation of pelargonidin-3-glucoside (Salvatierra et al., 2013). Similar results supporting the negative regulation of anthocyanins by *MYB1* were found in *F.* × *ananassa*, since the overexpression of *FaMYB1* led to a downregulation of *LAR* and *UFGT* and the subsequent reduction in the total anthocyanins content (Kadomura-Ishikawa et al., 2015b).

Afterwards, the most studied TF in strawberry, the key positive regulator of the anthocyanin biosynthesis *MYB10*, was characterized. The role of *MYB10* regulating anthocyanin biosynthesis in strawberry was first reported by stable overexpression in *F.* × *ananassa*, which resulted in an increase of the anthocyanins levels in fruits, flowers, leaves and roots (Lin-Wang et al., 2010). After that, a deeper characterization was made by transient silencing *FaMYB10* in *F.* × *ananassa* (Medina-Puche et al., 2014) and by stable overexpression in *F. vesca* (Lin-Wang et al., 2014), supporting its role promoting anthocyanin biosynthesis. Thus, *MYB10* downregulation produces fruits with white flesh and skin, while overexpression leads to a greater accumulation of pelargonidin-3-glucoside and cyanidin-3-glucoside. These changes are the consequence of an altered expression of many genes related to flavonoid biosynthesis, transport and regulation, like *PAL*, *CHS*, *CHI*, *F3H*, *DFR*, *ANS*, *UFGT* and *RAP/GST1* (GST transporter) (Lin-Wang et al., 2014; Medina-Puche et al., 2014), being the promoters of *CHS*, *UFGT*, *DFR* and *MYB10* itself directly regulated by MYB10 (Lin-Wang et al., 2014; Li et al., 2020). However, the relationship between MYB10 and MYB1 needs further analyses. Thus, despite dual-luciferase assays have shown that they could regulate each other (Lin-Wang et al., 2014), no significant differences in gene expression for *MYB10* have been observed after *MYB1* overexpression/silencing and vice versa (Medina-Puche et al., 2014). Remarkably, despite the importance of MYB10 in the regulation of anthocyanin biosynthesis, no further information about the direct target genes of this TF has been clarified yet, besides those previously mentioned genes.

Different genome-scale DNA analyses have identified allelic variation in *MYB10* as the main cause producing skin and flesh color variations in different species of *Fragaria* genus in nature (Hawkins et al., 2016; Castillejo et al., 2020; Manivannan et al., 2021). In particular, four different polymorphisms in *MYB10* have been described explaining the lack of anthocyanin in fruits from different accessions of *F. vesca* so far: (1) a G35C (W12S) SNP (Hawkins et al., 2016; Zhang et al., 2017), (2) an insertion at the third exon (LTR retrotransposon), (3) a single nucleotide insertion at position 329, and (4) a large deletion in chromosome 1 that removes *FvMYB10* (Castillejo et al., 2020). In *F.* × *ananassa* cv Camarosa, its main dominant homoelog is *FaMYB10-2*, which is also the allele with the highest genetic variation. In this case, the genotypes that present red-fleshed fruits have a large transposon insertion (~23kb) in the *FaMYB10-2* promoter that induces a higher expression of *MYB10*, probably contributing with some putative regulatory cis-elements to the promoter that were identified, such as ABA-, MeJA- and sugar-responsive elements, as well as MYB binding motifs and enhancers (Castillejo et al., 2020). In the same study, a new allele of *MYB10* was identified in *F. chiloensis* that also produces a premature stop codon due to an 8-bp insertion, generating a truncated version of the protein that lacks 54 amino acids at the C-terminal domain and that correlated with all white-fruited genotypes analyzed. The same polymorphism has been also described in the white-fruited *F.* × *ananassa* cv Snow Princess, which impairs the interaction of this truncated version of MYB10 with the WD40-repeat protein FaTTG1 (Wang et al., 2020). Interestingly, all white-fruited genotypes found in nature that have been characterized so far presented allelic variations in *MYB10*, supporting a convergent/parallel evolutionary mechanism to control anthocyanin biosynthesis since mutations in other regulators that lead to a general lack of flavonoids/anthocyanins in the plant could be detrimental due to their biological role against different stresses (Luo et al., 2018; Castillejo et al., 2020).

Besides *MYB1* and *MYB10*, other MYB TFs involved in the regulation of the phenylpropanoid pathway have been characterized or proposed as candidate regulators. Among them, *FvMYB79* transient overexpression and silencing up- and downregulate the expression of *MYB10*, *CHS*, *CHI*, *DFR* and *UFGT* (Cai et al., 2022). Another MYB TF, *FaMYB63*, which is related to the biosynthesis of phenylpropanoid-derived volatiles as we will discuss later, is also able to directly regulate *MYB10* expression in dual-luciferase assays (Wang et al., 2022). Moreover, several bHLH TFs have been involved in this pathway to date. Among them, the stable silencing of *bHLH33* produced fruits with no phenotypic effect regarding anthocyanin accumulation in *F. vesca*, probably due to redundancy with other bHLH TFs. However, it has been shown that FvbHLH33 strongly induces FvMYB10 regulatory activity upon *DFR* and *UFGT* promoters in transient transactivation assays, and that this activation could be reduced when FvMYB1 was co-transformed (Lin-Wang et al., 2014). Its homeolog in *F.* × *ananassa* (FabHLH33), described as a candidate for the regulation of PAs biosynthesis (Schaart et al., 2013), is able to physically interact with many members of the MYB family considered as either PA or anthocyanin biosynthesis regulators (Xu et al., 2021), as well as with FaTTG1 (Schaart et al., 2013). Another bHLH-like gene involved in this pathway is *PACLOBUTRAZOL RESISTANCE 1* (*FaPRE1*), an atypical HLH transcription regulator characterized by the absence of the basic domain with DNA-binding activity (Medina-Puche et al., 2019). Thus, *FaPRE1* transient silencing leads to a downregulation of *FaMYB10* and several genes of the phenylpropanoid pathway, i.e. *CHS*, *F3H*, *DFR*, a putative *anthocyanidin 3’-O-beta-glucosyltransferase* (*3´GT*) and *RAP/GST1*, probably through the regulation of *FaMYB10* (Medina-Puche et al., 2019). Consistent with these changes in gene expression, stable overexpression of *FaPRE1* increased the content of anthocyanins in leaves and petioles (Medina-Puche et al., 2021).

Other MBW-forming proteins reported to play a role in the phenylpropanoid pathway include the complex constituted by the paralogs FaMYB9 and FaMYB11, and FabHLH3 (ortholog to *F. vesca* FvH4_2g23700) and FaTTG1, which orthologs in Arabidopsis, i.e. AtTT2, AtTT8 and AtTTG1, respectively, physically interact and regulates PA biosynthesis (Walker et al., 1999; Nesi et al., 2000; Nesi et al., 2001; Schaart et al., 2013; Xu et al., 2021). In strawberry, the expression of *FaMYB9* and *FaMYB11* highly correlates with those of *F3’H*, *ANS*, *ANR* and *LAR*, as well as with the total PAs content in unripe fruits (Schaart et al., 2013). This study also suggests other TFs, i.e FaMYB5, the truncated version of FabHLH3, FabHLH3Δ and FaMYC1 as regulators of PA biosynthesis. Thus, interaction assays showed that FaMYB5, whose ortholog in Arabidopsis has an inhibitory role in the regulation of PAs biosynthesis, is also able to interact with FabHLH3. Similarly, the truncated FabHLH3Δ protein might have a negative regulatory role competing with other functional bHLHs in its interaction with FaMYB1, FaMYB5, FaMYB9 and FaMYB11. Finally, FaMYC1 might be also involved in PAs biosynthesis as its gene expression correlates with PAs content (Xu et al., 2021) and it interacts with FaTTG1 and FabHLH33 (Schaart et al., 2013). Some of these phenylpropanoid/PAs-related genes are also regulated by FaGAMYB. Thus, *FaMYB10*, *FaMYB1* expression is downregulated in *FaGAMYB-*RNAi fruits, while *FaMYC1* and *FaTTG1* are upregulated, which is also accompanied by a secondary metabolic profile characteristic of early stages of fruit ripening, i.e., lower anthocyanins (pelargonidin-3-glucoside and cyanidin-3-glucoside) and hydroxycinnamic acid derivatives, and higher PAs contents (Vallarino et al., 2015).

More components of the regulatory MBW complexes have been recently identified in the woodland strawberry. Among them, FvMYB3, FvMYB21, FvMYB22, FvMYB45, FvMYB64, FvMYB77 and FvMYB105 have been proposed as putative regulators of PAs biosynthesis, while FvMYB41 was associated with anthocyanin accumulation based on their phylogenetical relationship with MYB TFs involved in flavonoid biosynthesis in other species and the correlation of their expression with the accumulation of these compounds during fruit development and ripening (Xu et al., 2021). Protein interaction and transactivation assays demonstrated that several of the identified MYB TFs interact with bHLH proteins and promote the expression of *CHS2* and *DFR2* as well as PAs biosynthesis. In particular, FvMYB3, FvMYB9, FvMYB11 FvMYB21, FvMYB22, FvMYB41, FvMYB64, FvMYB75 and FvMYB105 were able to form complexes with FvbHLH3 (FvH4_2g23700), FvbHLH33, and FvMYC1. Furthermore, all those MYBs, as well as FvMYB45 and FvMYB77, but with the exception of FvMYB75, could bind to the *CHS2* promoter. In the case of *DFR2* promoter, FvMYB11 and FvMYB21 could bind it by themselves; FvMYB10 and FvMYB75 could bind it although only in the presence of FvMYC1; and FvMYB22, FvMYB64 and FvMYB105 also required the presence of coregulators, in particular FvbHLH3, FvbHLH33, or FvMYC1. Moreover, when these three MYBs, *FvMYB22*, *FvMYB64* or *FvMYB105*, were transiently co-expressed with *FvbHLH33*, they also induced *CHS2* and *DFR2* gene expression and PAs accumulation in strawberry fruits (Xu et al., 2021). All this data supports an extraordinarily complex regulation of the phenylpropanoid pathway, in which a high number of TFs may form heterocomplexes to fine-tune the biosynthesis of polyphenolic compounds.

### Other TFs involved in polyphenol biosynthesis in strawberry

As previously mentioned, *FaRIF* constitutes a major regulator of strawberry fruit ripening, playing a key role in phenylpropanoid biosynthesis. Thus, FaRIF regulates many structural genes like *PAL1, PAL2*, *C4H*, *4CL2*, *CHS1*, *CHI2*, *F3H, ANS, UFGT1* and *UFGT2*, as well as other anthocyanin-related regulators such as *PRE1*. Remarkably, transcriptomic data in *FaRIF-*silenced lines showed that *FaRIF* downregulation produces a drift in the metabolic flux from the flavonoid branch to the monolignol pathway, resulting in a reduction in the anthocyanin content, but an increase in the precursors of lignin biosynthesis, such as coumaric acid and the hexose derivatives of the coumaric, caffeic and ferulic acids. Consequently, these changes produce a higher accumulation of lignin content in the fruit receptacle (Martín-Pizarro et al., 2021).

MADS-box TFs are also involved in the synthesis of polyphenolic compounds. Hence, *FaMADS9* silencing results in an alteration in their content. In particular, green fruits showed a lower content of ellagitannins, galloyl, quercetin and kaempferol derivatives, and an increase levels of PAs compared to the control. At the red stages, the pelargonidin derivative compounds were increased, while the cyanidin derivatives were reduced, probably due to the downregulation of *F3’H* in white receptacles (Vallarino et al., 2020). Other phenylpropanoid-related genes differentially expressed in *FaMADS9-*RNAi lines included *CHS1*, *CHI1, CHI3*, *F3H*, *MYB10*, *RAP* and other three GST transporters, which were upregulated at the white stage. Furthermore, *PAL1, PAL2*, *C4H*, *4CL*, *F3H* (Vallarino et al., 2020) and *CHS* (Seymour et al., 2011) were downregulated at the ripe stage. Other MADS-box, *FaSHP*, positively regulates the anthocyanin biosynthesis since transient *FaSHP*-silenced fruits present a downregulation in the expression of *PAL*, *CHS*, MYB1 *MYB10* and *MADS9*, resulting in a decrease in the anthocyanin content, and higher levels of unripe-characteristics compounds such as caffeic acid derivatives and ellagitannins (Daminato et al., 2013). Besides these MADS-box regulators that play a positive role in the regulation of the flavonoid pathway, a negative regulator has also been identified. In particular, *FaMADS1a* expression decreases during ripening and its overexpression results in a delayed ripening and a reduction in the content of anthocyanin due to the downregulation of structural genes such as *FaPAL6*, *FaCHS*, *FaDFR* and *FaANS* (Lu et al., 2018). It has been recently reported the role of miR5290 in the regulation of *FaMADS1a.* Thus, miR5290 expression, which is induced during ripening by ABA, represses *FaMADS1a*, thus releasing its negative regulatory effect on anthocyanin biosynthesis (Chen et al., 2022).

Other families of TFs have also been shown to regulate strawberry fruit color. *FaRAV1* is an AP2/ERF TF, which transient overexpression promotes anthocyanin accumulation due to the promotion of *CHS*, *CHI*, *F3H*, *DFR*, *ANS* and *GT1* expression both directly and indirectly, as it is also a direct positive regulator of *MYB10* (Zhang et al., 2020). Another TF, *FvTCP9*, not only regulates ABA biosynthesis, as previously discussed, but also promotes anthocyanin biosynthesis, supported by transient overexpression and silencing assays that resulted in the upregulation and downregulation respectively of structural genes such as *C4H*, *4CL*, *CHS*, *CHI*, *F3H*, *DFR*, *ANS, UFGT* and the TFs *MYB1* and *MYB10* (Xie et al., 2020). Furthermore, FvTCP9 interacts with FvMYC1, so it has been proposed that FvTCP9 might regulate this metabolic pathway in a direct way as a partner of FvMYC1 and indirectly promoting ABA biosynthesis (Xie et al., 2020).

### TFs involved in flavonoid biosynthesis in response to abiotic/biotic stimuli

Environmental conditions such as light and temperature are known to modulate flavonoid biosynthesis in fruits (Takos et al., 2006; Kadomura-Ishikawa et al., 2015a; Xu et al., 2018; Zhang et al., 2018a), and several regulators of these processes have been described recently. Among them, it has been reported the role of the bZIP TF *FvHY5*, and *FvbHLH9* in the regulation of anthocyanin biosynthesis in response to light (Li et al., 2020). Thus, both TFs are induced by light in strawberries (Xu et al., 2018) and their transient overexpression promote anthocyanin biosynthesis, interacting to form an heterodimeric complex that directly binds to the promoters of *CHS* and *DFR* (Li et al., 2020). In a similar way, the B-Box TF FaBBX22 is able to form heterodimers with FaHY5 and regulate *PAL*, *ANS*, *F3’H*, *UFGT* and *RAP* gene expression in a light-dependent manner, resulting in a higher accumulation of anthocyanins (Liu et al., 2022).

Low temperatures result in a reduction in the anthocyanin content in strawberry. It has been recently reported the role of MITOGEN-ACTIVATED PROTEIN KINASE3 (FvMAPK3), which mediates the response to low temperature phosphorylating MYB10 and CHS proteins. This modification results in a reduction of MYB10 transcriptional activity and the proteasome-mediated degradation of CHS1, therefore negatively regulating the anthocyanin biosynthesis (Mao et al., 2022).

The role of flavonoids has been well described regarding plant defense (Shah and Smith, 2020), so usually biosynthetic genes from this pathway are analyzed when studying plant response against pathogens. In this context, *FaWRKY11*, which is a positive regulator of fruit resistance against *Botrytis cinerea*, can also promote the expression of *MYB1* and *MYB10*, although fruits in which this TF were transiently overexpressed or silenced did not display any evident color phenotype (Wang et al., 2021). In a similar way, *FaWRKY1*, another positive regulator of plant resistance, is able to induce *GST* in *A. thaliana* (Encinas-Villarejo et al., 2009), while *FaWRKY25* is a negative regulator of *CHI2* and *CHI3* (Jia et al., 2021). Besides the role of these WRKY TF, a bZIP-like protein, *FvbZIP46* has also been shown to positively induce fruit resistance against *B. cinerea* and positively regulate *CHI2*, *CHI3* and *CHI4* gene expression (Lu et al., 2020a). Although these genes are known to contribute to plant defense, the effect of misregulating *FvbZIP46* on anthocyanin production has not been reported yet.

## Regulation of sugar metabolism

Sugar content in strawberry fruits is one of the most important traits as it is the main determinant of consumer preferences (Yan et al., 2018). It does not only affect the sweetness perception, but the ratios between sugars and organic acids play an important role in the final flavor (Fait et al., 2008; Fan et al., 2021). Glucose, fructose and sucrose are the main soluble sugars in strawberry, although sucrose is the sugar with the highest increase during ripening (Fait et al., 2008). Sucrose biosynthesis starts in the cytosol with the combination of fructose 6-phosphate and UDP-glucose to form sucrose 6-phosphate by the sucrose phosphate synthase (SPS). In the next step sucrose 6-phosphate is dephosphorylated by sucrose phosphate phosphatase (SPP) to form sucrose, the principal sugar transported from photosynthetic to sink tissues. Inside sink cells, sucrose can be hydrolyzed either to glucose and fructose by invertases in an irreversible reaction, or to fructose and UDP-glucose by sucrose synthase (SUS), which catalyses a reversible reaction instead (Stein and Granot, 2019). Moreover, sugars are known to be important regulators of many processes as they present signaling properties, including strawberry fruit ripening (Jia et al., 2013; Jia et al., 2016).

Among the TFs described to play a role in the regulation of sugar metabolism in strawberry fruits (**Figure 1C**), FaGAMYB has been shown to promote sugar biosynthesis. Thus, *FaGAMYB*-silenced fruits displayed a decreased sucrose content compared with the control, consistent with the downregulation of *FaSPS1, FaSPS2* and *FaSPS3*, and the upregulation of *FaSUS* (Vallarino et al., 2015). Another MYB TF, *FaMYB44.2* has been shown to play an important negative role. Its transient overexpression leads to a decrease in the content of glucose, fructose and sucrose, which is explained by the altered expression of genes related to sucrose biosynthesis, degradation and transport, such as *FaSUS1*, *FaSPS1/2/3* and *FaSUT1*. Besides, other genes that can alter sucrose metabolism indirectly, like hexokinase 2 (*FaHXK2*) and trehalose-6-phosphate synthase 7 (*FaTPS7*) were also differentially expressed. Furthermore, FaMYB44.2 directly interacts with *FaSPS3*, *FaSUS1* and *FaHXK2* promoters and forms protein complexes with other members of its family, i.e., FaMYB44.1, FaMYB44.3, as well as with phenylpropanoid-related TFs such as FaMYB1, FabHLH3 (FvH4_2g22150, which is different to the previously named FabHLH3), FabHLH33 and FaTTG1. In the current proposed model, FaMYB44.2 and FabHLH3 form a complex that negatively regulates *FaSPS3* expression and therefore sucrose biosynthesis. However, Wei and collaborators also showed that this repression is impaired by FaMYB10, which competes for FabHLH3 binding during ripening (Wei et al., 2018). Despite the role in the regulation of sugar metabolism, the misregulation of *FaMYB44.2* also results in an alteration of different organic acids and volatile compounds as well as the expression of genes involved in other ripening-related processes, such as anthocyanin biosynthesis (*FaMYB1* and *FaMYB10*), hormone signaling (*FaPYL1*, *FaJAZ1* or *FaARF6B*), and general ripening regulation (*FaGAMYB*).

FaRIF was also reported to be involved in sugar metabolism (Martín-Pizarro et al., 2021). Thus *FaRIF-*downregulated fruits were also affected in their sugar metabolism, producing a higher accumulation of glucose and fructose, and a reduction in the sucrose content, which is supported by the downregulation of *FaSPS1* and the upregulation of *FaSUS1*. Furthermore, genes encoding glycolytic and fermentation enzymes are downregulated by FaRIF, supporting an important role in the regulation of the aerobic/anaerobic balance that changes during strawberry fruit ripening (Wang et al., 2017; Martín-Pizarro et al., 2021). Finally, *FaMADS9* also positively promotes sugar accumulation during ripening since RNAi fruits for this TF showed a reduction of Brix content as the consequence of a reduced content of sucrose, glucose and fructose (Vallarino et al., 2020). Furthermore, this study showed that *FaMADS9* regulates starch degradation, which is important during strawberry fruit development (Souleyre et al., 2004). Thus, the levels of maltose and isomaltose, disaccharides produced by the hydrolysis of starch, and some amylases-encoding enzymes were altered in *FaMADS9*-RNAi fruits, being therefore disrupted in these fruits the degradation of starch to fuel the fruit growth and ripening (Vallarino et al., 2020).

Finally, two members of the bZIP TF family have been reported to positively regulate sugar accumulation. Hence, the heterologous overexpression of *FvbZIP11* in tomato fruits produced a higher content of total soluble solids and sugars (Zhang et al., 2022b) while transiently overexpressing *FabZIPs1.1* in strawberry fruits induced a greater accumulation of sucrose (Chen et al., 2020).

## Regulation of volatile compounds biosynthesis

Volatile organic compounds (VOCs) influence strawberry flavor and aroma, essential traits for fruit quality. More than 360 VOCs have been identified in strawberry fruit, but it is generally considered that only a part of them are able to influence the organoleptic properties. These compounds constitute a diverse group, including esters, aldehydes, alcohols, ketones, terpenes, furanones and alkanes. During strawberry ripening most acids, esters, furan, ketones, lactones and terpenes increase their levels, while most alcohols, aldehydes, alkanes, and furanones exhibit a decrease (Lu et al., 2020b). Among all groups, esters are the most represented compounds in ripe fruits (Song and Forney, 2008), ranging from 25% to 90% of total volatiles, and contributing as the main source of fruity and floral odors (Yan et al., 2018) and with a sweetness-enhancing ability (Fan et al., 2021). In contrast, C6 aldehydes have been identified as the major compounds in immature fruits (Song and Forney, 2008).

### C6 volatiles and esters biosynthesis

Many volatile compounds, including esters, are derived from fatty acids (FAs) through the LOX pathway, which starts with the transformation of linoleic (18:2) and linolenic (18:3) acids into their hydroperoxide derivatives by β-oxidation carried out by lipoxygenases (LOXs) (Lu et al., 2022). Hydroperoxide isomers can be further metabolized to aldehydes by hydroperoxide lyase (HPL), which in turn are reduced by alcohol dehydrogenase (ADH) to form alcohols, that can finally be the substrate of alcohol acyl transferase (AAT) to produce esters (Lu et al., 2021).

Besides the role regulating PAs content, the paralogs *FaMYB9* and *FaMYB11* also regulate the content of volatile C6 and esters compounds respectively (Lu et al., 2020; Lu et al., 2021). Thus, *FaMYB9*, whose expression decreases during the ripening process, is a positive regulator of C6 volatiles since its silencing leads to a general reduction of these compounds (Lu et al., 2020). Specifically, *FaMYB9* regulates the content in hexanal and (*E*)-2-hexenal, which can contribute to the grassy flavor of unripe fruits (Larsen and Poll, 1990; Du et al., 2011), and in methyl isovalerate, which contributes to fruity notes (Alstrup et al., 2020). Lu and collaborators also found that FaMYB9 protein physically interacts with FaLOX5, regulating as well its expression and that of other LOX pathway genes. Similarly, FaMYB11 also induce *FaLOX5* expression by directly binding to its promoter. Furthermore, FaMYB11 also promotes the expression of *FaADH* and *FaAAT* among other genes of the FA biosynthetic pathway. Therefore, *FaMYB11* transient up- and downregulation modify the volatile composition, mainly in the content of aldehydes and esters (Lu et al., 2021). *FvMYB10* is also able to regulate esters production. Hence, stable *FvMYB10* overexpression lines have been shown to produce fruits with a higher content of the esters butyl and hexyl acetate, which level contributes to consumer preferences (Klee and Tieman, 2018). *MYB10* overexpression also leads to increased levels of ethyl butanoate, but a reduction in the content of octyl acetate. In contrast, *FvMYB10-*silenced fruits only showed a significant increase of 2-heptanone, which also contributes to flavor and consumer preferences (Lin-Wang et al., 2014; Klee and Tieman, 2018).

### Furanones biosynthesis

In strawberry, furanone-derived volatiles are mainly represented by 4-hydroxy-2,5-dimethyl-3(2H)-furanone (HDMF, furaneol) and 2,5-dimethyl-4-methoxy-3(2)H-furanone (DMMF, mesifurane), which are considered to generate a caramel-like aroma (Larsen and Poll, 1990) and contributes to the sweetness of the fruit (Fan et al., 2021). Although the biosynthetic pathway has not yet been totally elucidated, 4-hydroxy-5-methyl-2-methylene-3(2H)-furanone (HMMF) has been identified as the immediate precursor of HDMF, catabolized by the enzyme quinone oxidoreductase (QR). In this context, a MYB TF, FaMYB98, has been identified in a yeast-one-hybrid screening as a direct regulator of *FaQR* transcription. Moreover, it has been found that *FaQR* expression synergistically increases when FaMYB98 forms a complex with the Ethylene-Response Factor protein FaERF9, which depends on the former to promote *FaQR* transcription and the biosynthesis of furaneol (Zhang et al., 2018b). Furthermore, other ripening-related TFs have been found to regulate *FaQR* expression. Thus, *FaQR* is downregulated when *FaMADS9*, *FaSHP*, *FaPRE1* and *FvTCP9* are silenced, supporting a positive role of these regulators on the biosynthesis of furaneol (Seymour et al., 2011; Daminato et al., 2013; Medina-Puche et al., 2019; Xie et al., 2020).

### Eugenol biosynthesis

Eugenol is another important volatile compound contributing to strawberry fruit aroma, and it derives from the phenylpropanoid pathway (Medina-Puche et al., 2015). Eugenol biosynthesis starts with the transformation of feruloyl-CoA into coniferyl aldehyde by the cinnamoyl-CoA reductase (CCR). Coniferyl aldehyde is then reduced by the cinnamyl alcohol dehydrogenase (CAD) to generate coniferyl alcohol, which is converted to coniferyl acetate by a coniferyl alcohol acetyltransferase (CAAT), and finally transformed to eugenol by the Eugenol Synthase (EGS) (Rastogi et al., 2013). Several TFs have been identified as regulators of eugenol biosynthesis. Among them, the MYB TF FaEOBII positively regulates eugenol production, as its silencing produces a reduction of its content due to the downregulation of the structural genes *FaCAD1* and *FaEGS2.* A transactivation assay also showed that FaEOBII directly binds to FvCAD1 promoter. Interestingly, *FaEOBII* is in turn positively regulated by FaMYB10 (Lin-Wang et al., 2014; Medina-Puche et al., 2015). Thus, FaMYB10 also contributes to the regulation of the branch of the phenylpropanoid pathway responsible for the biosynthesis of these volatile compounds since, besides *FaEOBII*, it regulates the expression of *CCR* and *CAD* genes (Medina-Puche et al., 2014). Another MYB-like TF involved in eugenol biosynthesis is *FaMYB63*, which is able not only to directly regulate structural genes of the pathway, i.e., *FaCAD1*, *FaEGS1*, and *FaEGS2*, but also indirectly positively regulating *FaMYB10* and *FaEOBII* expression (Wang et al., 2022).

Another eugenol-related TF is FaDOF2, a protein belonging to the plant-specific DOF (DNA binding one zinc finger) family. FaDOF2 positively regulates *FaEOBII* and *FaEGS2* gene expression, probably by direct binding to their promoters, as they present several binding sites that can be recognized by FaDOF2 (Molina-Hidalgo et al., 2017). Interestingly, *FaDOF2* and *FaEOBII* constitute a positive feedback loop, since *FaDOF2* expression is also positively modulated by *FaEOBII*. Furthermore, FaDOF2 interacts with FaEOBII generating a complex that fine-tunes the expression of genes involved in eugenol production. *FaPRE1* has also been described to positively regulate the expression of the regulator *FaEOBII* as well as that of the structural genes *FaCAD1* and *FaEGS2* and two alcohol acyl transferases involved in esters biosynthesis (*FaAAT1-2*) (Medina-Puche et al., 2019). Finally, FaRIF is also involved in this pathway, since it promotes the expression of eugenol-related genes, both in a direct way by inducing the *FaEGS2* expression, and indirectly through the activation of *FaEOBII* and *FaDOF2* (Martín-Pizarro et al., 2021). Furthermore, FaRIF has also been suggested as a regulator for the production of the terpenic volatile compounds linalool and nerolidol, since the responsible gene for their biosynthesis, *NEROLIDOL SYNTHASE1* (*FaNES1*), was downregulated in *FaRIF* silenced fruits (Martín-Pizarro et al., 2021).

In summary, the biosynthesis of different aroma-related compounds is regulated by a complex gene regulatory network (GNR) involving different types of TFs (**Figure 1D**).

## TFs-mediated regulation of fruit softening

Fruit softening is a complex process that includes cell wall disassembly and degradation of the middle lamella, leading to a lower cell-to-cell adhesion. The primary cell wall is mainly formed by a cellulose microfibril network, a glycan matrix, and a pectin network, held together and cross-linked with other wall components (Posé et al., 2011). Pectin is the most abundant class of macromolecule within the primary cell wall and the middle lamella matrixes, and its solubilization is known to be the most consistent feature during strawberry fruit softening (Posé et al., 2011). The role of many enzymes during cell wall disassembly has been characterized specifically regarding fruit ripening processes. The activity of enzymes like pectate lyases (PL), polygalacturonases (PG), pectin esterase (PE), pectin methylesterase (PME), β-1,4-glucanases (EGase), expansins (Exp), β-xylosidases (Xyl), β-galactosidase (β-Gal), and α-arabinofuranosidases (Ara) modify different polysaccharides, especially matrix glycans and pectins (Posé et al., 2011).

A number of TFs have been deeply characterized in their role regulating cell wall composition (**Figure 1E**). Among them, a WRKY-type protein, *FvWRKY48*, whose expression increases during strawberry fruit ripening, regulates the chemical properties of the cell wall during this process (Zhang et al., 2022a). Thus, this TF regulates the reduction of homogalacturonan (HG) pectin polymer, since stable overexpressing and silencing lines produce fruits that present lower and higher content respectively in the middle lamella and tricellular junction zone. These changes are reflected in altered fruit firmness and ripening progress of those transgenic lines and are explained by the positive regulation of three *PL* genes and one *β-Gal* by FvWRK48. Furthermore, yeast-one-hybrid, EMSA, and ChIP-qPCR assays identified a direct interaction of FvWRKY48 in the promoter region of one of the *PL* genes, *FvPLA*, whose overexpression and silencing is able to mimic the HG content phenotype in the *FvWRKY48* misexpression.

*FaRIF* has also been found to be a central regulator of cell wall composition. Stable *FaRIF-*silenced and overexpression lines develop firmer and softer receptacles respectively, supporting a role of this TF in promoting fruit softening during ripening. The general role of FaRIF in the regulation of cell wall composition is confirmed by a transcriptome analysis in fruits from those transgenic lines that showed a misregulation of cell wall degradation-related genes such as *FaXYL3*, *FaPL2*, *FaPL3*, *FaPL4*, *FaGHB15* (EGase), and the PGs *FaPG1* and *FaADPG2*. Moreover, the expression of enzymes responsible for modifying the cell wall coding genes was also altered, including *FaEXP1, FaEXP2, FaEXP3*, *FaPME38*, *FaPME39* and different *FaAGPs*, which encodes for arabino galactan-proteins (Martín-Pizarro et al., 2021). Besides FaRIF, another NAC protein, FcNAC1 has been shown to directly induce *PL* expression, although its function in cell wall remodeling has not been studied yet (Carrasco-Orellana et al., 2018). Another direct regulation has been described for *FvMYB79*, which besides regulating the expression of phenylpropanoid-related genes, binds to the promoter of *FaPME38* activating its expression (Cai et al., 2022). Furthermore, the expression of *PME*, *EXP*, *PL*, *PG* and *EGase* genes was significantly downregulated in *FvMYB79-*RNAi fruits, and upregulated when this TF was overexpressed (Cai et al., 2022). Similar transient experiments allowed to identified cell wall-related genes differentially expressed in *FvTCP9* silenced and overexpression lines, including *β-Gal1/2/3* and *EXP1/2/5* (Xie et al., 2020).

Among the MADS-box proteins involved in strawberry fruit softening, *FaMADS9* might promote this process during ripening, since its downregulation increased fruit firmness in the work of Seymour and collaborators (Seymour et al., 2011), but not in Vallarino’s study (Vallarino et al., 2020). Again, and as previously mentioned, this difference might be the consequence of the possible off-target effects over other related MADS-box genes in Seymour’s work. Nevertheless, both studies identified differences in the expression of genes related to cell wall modifications in *FaMADS9*-RNAi fruits, which showed an upregulation of *PE2* (Seymour et al., 2011) and *PG2* (Vallarino et al., 2020), and a downregulation of cellulase, *PL1* and *PE1* in red fruits (Seymour et al., 2011), and an upregulation of *PG1* and *PG2* in white fruits (Vallarino et al., 2020), supporting a role of this TF in the modulation of the cell wall composition. Besides the role of the MADS-like protein *FaMADS9*, transient silencing and overexpression of *FaSHP* altered the expression of *PG1*, *PL* and *EG1* (Daminato et al., 2013), although its role in the regulation of fruit firmness or cell wall composition has not been clarified yet.

Finally, *FaPRE1* has also been proposed to control cell wall metabolism. Thus, *FaPRE1* downregulation negatively regulates *FaPG1* and *FaRGlyaseI* and produces an upregulation of a number of cell wall genes whose transcription is usually higher in immature fruits (Medina-Puche et al., 2019). Although *FaPRE1* expression is receptacle-specific, its ectopic overexpression leads to an elongation of vegetative organs, probably due to the regulation of many genes that encode cell wall-modifying enzymes (Medina-Puche et al., 2021). However, the role of *FaPRE1* in the regulation of fruit softening during strawberry fruit ripening requires further investigation.

## Candidate regulators of strawberry development and ripening

A number of transcriptome studies have been performed in order to identify whole strawberry TF families. These studies are usually accompanied by a brief characterization of the family and their putative role in the regulation of different processes, like their regulatory role in hormone metabolism, flavonoid biosynthesis or response against pathogens. In these studies, different members of these families have been proposed as candidate regulators of specific traits related to fruit ripening. For example, the NAC TF family was studied in *F.* × *ananassa* regarding its putative role in fruit development and ripening (Moyano et al., 2018). Thus, the expression of *FaNAC006*, *FaNAC021*, *FaNAC022*, *FaNAC035* (*FaRIF*) and *FaNAC042* is induced during fruit development and ripening. Furthermore, their expression was downregulated after fruits were treated with 1-Nordihydroguaiaretic acid (NDGA), an inhibitor of ABA biosynthesis, supporting their potential role as regulators of strawberry fruit ripening, although only *FaRIF* has been functionally validated so far among these candidate genes (Martín-Pizarro et al., 2021). Furthermore, *FaNAC022* and *FaNAC042* were suggested as regulators of vascular tissue and secondary cell wall development, and *FaNAC006* and *FaNAC092* as fruit senescence regulators (Moyano et al., 2018).

Other members of MBW complexes have been proposed as putative regulators of anthocyanin biosynthesis based on their ripening-related pattern expression and function of their putative orthologs, such as *FabHLH17*, *FabHLH25*, *FabHLH27*, *FabHLH29*, *FabHLH40*, *FabHLH80* and *FabHLH98* from the bHLH family (Zhao et al., 2018), and *FaMYB28*, *FaMYB54* (*MYB1*) and *FaMYB576* from the MYB family (Liu et al., 2021), as well as *FvMYB33* as a possible PAs biosynthesis regulator (Shulaev et al., 2011). In a similar way, the GRAS TFs were proposed as regulators of strawberry fruit ripening. In particular, *FvGRAS27* was also proposed as a regulator of anthocyanin accumulation and *FvGRAS54* as a general regulator of ripening (Chen et al., 2019). Regarding the TCP family, *FvTCP12* and *FvTCP17* could also be involved in regulating ripening-related processes as their expression shows an increasing pattern during ripening and are highly induced after ABA treatment (Wei et al., 2016). Finally, *FaERF3*, *FaERF6* and *FaERF71a* were also proposed as ripening regulators due to their expression pattern (Sánchez-Sevilla et al., 2017). In conclusion, there are a large number of putative regulators of strawberry fruit ripening which role has not been validated yet, so their study will shed light on their contribution to this process.

## Conclusion and future perspectives

In this work, we have summarized the role of a large number of TFs that have been studied in relation to the control of different processes related to strawberry fruit ripening to date. However, besides all this knowledge acquired so far, the precise regulation of each specific process involved during strawberry fruit development and ripening is still poorly understood. One explanation is the complexity itself of strawberry as a model plant, which hinders, and even prevents in the case of the octoploid species, to perform genetic studies to understand the relationship between those regulators. Therefore, most studies are based on the phenotypical and molecular characterization of either RNAi or overexpression stable transgenic lines, or on transient assays, where resulting transcriptome changes can be followed. However, no study has been performed so far to identify the direct targets and consensus DNA-binding sequences of any TF genome-wide with assays like DAP (DNA Affinity Purification) or ChIP (Chromatin Immunoprecipitation) sequencing, with the exception of a targeted analysis by ChIP-qPCR to identify the direct regulation of *PLA* by FvWRKY48 (Zhang et al., 2022a) and an *in vitro* DNA binding assay that identified the consensus sequence of FaDOF2 (Molina-Hidalgo et al., 2017). This is the consequence of the complexity of the ChIP methodology in general and in strawberry in particular, considering that stable tagged-TF overexpression lines in order to use efficient commercial antibodies are not easy to obtain, nor the ChIP protocol to optimize. On top of that, the polyploidy of *F.* × ananassa is another important challenge that complicates genomic studies. However, we believe that the *in vitro* approach DAP-seq will become a very useful and informative alternative that will be widely used in the identification of direct target genes of strawberry TFs in the near future. Finally, it has been recently and successfully applied the CRISPR/Cas9 genome-editing tool in strawberry plants (Zhou et al., 2018; Gao et al., 2020; Pi et al., 2021; Mao et al., 2022), including the octoploid species (Martín-Pizarro et al., 2019; Wilson et al., 2019). This tool brings new opportunities to deeply characterize TFs, avoiding misleading conclusions about their role that might arise in either knockdown assays or with spontaneous mutations. For example, the TFs RIPENING INHIBITOR (RIN), NON-RIPENING (NOR), and COLORLESS NON-RIPENING (CNR) have traditionally been considered master regulators of tomato fruit ripening (Seymour et al., 2013). However, CRISPR/Cas9 knockout lines for these TFs resulted in mutants where the developing fruits displayed a more subtle ripening phenotype than the original spontaneous mutants, which have been shown to be gain-of-function (*rin*) or dominant-negative (*nor* and *cnr*) mutations (Ito et al., 2017; Gao et al., 2019; Wang et al., 2019). These results question the role of just a few TFs as the upstream master regulators of fruit ripening, and suggest a more complex network of TFs underlying the control of this process in tomato, and probably in other species such as strawberry. This highlights the promise of CRISPR/Cas9 mediated dissection of molecular processes in crop species like strawberry and, together with the integration of transcriptome studies and ChIP-seq/DAP-seq analyses, will open a new horizon in the discovery and characterization of the regulatory networks that control strawberry fruit ripening.

## Author contributions

CS-G, DP, and CM-P conceived and wrote the manuscript. All authors contributed to the article and approved the submitted version.

## Funding

This work was supported by grants from the European Research Council (ERC-2014-Stg 638134 to DP), the Spanish Ministry of Science and Innovation (RTI2018-097309-A-I00 and PID2021-123677OB-I00 to DP) and the Junta de Andalucía (UMA20-FEDERJA-093 and POSTDOC21_00893 to CM-P). We thank Plan Propio from the University of Málaga for financial support.

## Acknowledgments

We thank Danelle K. Seymour for her suggestions to improve the manuscript.

## Conflict of interest

The authors declare that the research was conducted in the absence of any commercial or financial relationships that could be construed as a potential conflict of interest.

## Publisher’s note

All claims expressed in this article are solely those of the authors and do not necessarily represent those of their affiliated organizations, or those of the publisher, the editors and the reviewers. Any product that may be evaluated in this article, or claim that may be made by its manufacturer, is not guaranteed or endorsed by the publisher.
